# Communication across autism, ADHD, and non-clinical groups: examining autistic and ADHD traits from a dimensional perspective

**DOI:** 10.3389/fpsyg.2026.1865249

**Published:** 2026-08-28

**Authors:** Ramona Cardillo, Rachele Lievore, Enrico Toffalini, Giulia Crisci, Irene Cristina Mammarella

**Affiliations:** 1 Department of Developmental Psychology and Socialisation, University of Padua, Padua, Italy; 2 Department of General Psychology, University of Padua, Padua, Italy

**Keywords:** ADHD, adolescents, autism, children, communication, dimensional framework

## Abstract

**Introduction:**

Communication difficulties are common among youth with Autism Spectrum Disorder (ASD) and Attention-Deficit/Hyperactivity Disorder (ADHD). However, traits associated with ASD and ADHD are continuously distributed in the general population, raising the question of how communication differences are better described when considering both diagnostic categories and dimensional trait variation.

**Methods:**

This study included 493 Italian children and adolescents aged 7–16 years old (*M* = 11.35; *SD* = 2.64): 147 (126 boys) with ASD without intellectual disability, 105 (88 boys) with ADHD, and 241 (203 boys) non-diagnosed (ND) peers. Parents completed standardized measures assessing communication skills, ASD and ADHD traits.

**Results:**

Both clinical groups showed significant communication difficulties compared to ND peers, with large effect sizes (ASD-ND: *d* = −1.67; ADHD-ND: *d* = −1.00). In this dataset, communication outcomes were more parsimoniously modelled using continuous ASD and ADHD trait measures (BIC = 4151.39) than diagnostic group membership alone (BIC = 4324.75). Both trait dimensions were associated with communication challenges across clinical and non-diagnosed participants, although the association with ADHD traits was weaker.

**Discussion:**

These findings suggest that communication difficulties may be usefully described in relation to continuous ASD and ADHD trait variation, while preserving the clinical relevance of diagnostic categories.

## Introduction

Challenges with communication are widely recognized across various neurodevelopmental conditions (Bellani et al., 2011; Ferrara et al., 2020), being associated with functional, adaptive, and emotional consequences (Helland et al., 2022). These difficulties have been examined, especially in Autism Spectrum Disorder (ASD) and Attention-Deficit/Hyperactivity Disorder (ADHD), two of the most prevalent neurodevelopmental conditions. ASD affects approximately 1% of the population (Zeidan et al., 2022) while ADHD 5–7% (Zhong and Porter, 2024). Comorbidity between these conditions is common, with impairments in language and communication across both populations (Bellani et al., 2011; Korrel et al., 2017; May et al., 2018; Ferrara et al., 2020). Studies comparing youth with ASD and ADHD have shown similarities in their communication profiles (Geurts and Embrechts, 2008; Helland et al., 2012; Roselló et al., 2017; de la Torre Carril et al., 2021; Mammarella et al., 2022). Autistic children tend to experience more profound communication difficulties, but children with ADHD may also face challenges in both structural and pragmatic language (Dolata et al., 2022). Structural difficulties affect phonology, semantics, syntax and morphology, whereas pragmatic challenges refer to difficulties in the appropriate use of language during social interactions (Bishop and Baird, 2001; Geurts et al., 2004).

Communicative difficulties represent core features of ASD (American Psychiatric Association, 2013). Although structural language abilities can vary considerably among autistic children (Tager-Flusberg et al., 2005; Tek et al., 2014), difficulties in pragmatic language are consistently reported as a defining characteristic of their communicative profile (Parsons et al., 2017; Reindal et al., 2023). These challenges persist even in the absence of structural language impairments or intellectual disability (Young et al., 2005; Loukusa et al., 2007; Simmons et al., 2014; Sturrock et al., 2023) and are particularly evident in areas such as turn-taking, regulating the length and relevance of verbal contributions, and interpreting non-literal meanings (Volden and Phillips, 2010; Whyte and Nelson, 2015; Andrés-Roqueta and Katsos, 2017; Mammarella et al., 2022).

Although communication impairments are not included in the diagnostic criteria for ADHD, research has shown that children with this condition often experience challenges in expressive, receptive, and pragmatic language skills (Korrel et al., 2017; Carruthers et al., 2022; Kessler and Ikuta, 2023; Jepsen et al., 2024). This vulnerability to communication deficits has been explained from multiple perspectives (Carruthers et al., 2022). Several core symptoms of ADHD, such as not responding when spoken to or difficulty with conversational turns, may overlap with communicative difficulties, blurring the distinction between primary ADHD symptoms and secondary language-related manifestations (Green et al., 2014; Kuijper et al., 2017). However, these difficulties are often considered secondary to inattention and hyperactivity/impulsivity (Camarata and Gibson, 1999; Parks et al., 2021).

Despite their occurrence in clinical conditions, mild deficits in communicative competence may extend into the general population, where they are associated with functional and behavioral impairments (Skuse et al., 2009). These communicative difficulties may, in some cases, be underpinned by subclinical traits of neurodevelopmental conditions. Indeed, some authors argue that conditions such as autism (Happé and Frith, 2021) and ADHD (Levy et al., 1997; Ronald et al., 2021), may be better understood as existing along a spectrum. Subclinical autistic traits, for example, are associated with challenges in interpreting nonverbal cues essential for effective social communication (Landa et al., 1992; Ingersoll, 2010). Evidence suggests that language impairments may not differ substantially between autistic children and those who fall below the diagnostic thresholds (Reindal et al., 2023), indicating that even subclinical autistic traits may be linked to communication challenges. Similarly, ADHD-related traits predict pragmatic language difficulties in everyday contexts, even among the general population (Rints et al., 2015). Indeed, attentional and hyperactivity issues have been linked to poorer structural language development and reduced informativeness in conversations (Nilsen et al., 2015; Hawkins et al., 2016). Taken together, these findings support a dimensional view of neurodevelopmental traits and their association with communicative competence, suggesting that variation in language and pragmatic abilities reflects the continuous expression of ASD and ADHD related characteristics across typical and atypical development (Campbell and Skarakis-Doyle, 2011; Lancaster and Camarata, 2019). This perspective underscores the importance of considering neurodevelopmental traits, rather than categorical diagnoses alone, in communication research.

The debate over whether clinical symptoms reflect discrete categories (e.g., ASD, ADHD) or continuous dimensions (traits) is longstanding in clinical psychology (Widiger and Samuel, 2005; Kotov et al., 2017) but has only recently gained traction in neurodevelopmental research (Happé and Frith, 2020; Chown and Leatherland, 2021). Traditionally, dimensional perspectives have been explored using taxometric analysis, testing the continuity of relationships across variables (Meehl, 1995). However, taxometric methods require large representative samples with sufficient naturally occurring clinical cases to avoid “artificial admixture,” which can produce false or pseudo-taxa results (Grove, 1991; Ruscio and Ruscio, 2004). For instance, identifying ASD as a taxon with a 1% prevalence would demand over 3,000 general population participants to yield at least 30 ASD cases. Due to the impracticality of this approach for our study, we instead employed a method conceptually akin to taxometric analysis but adapted for use with separately recruited general and clinical samples (Mammarella et al., 2021; Carretti et al., 2022).

Building on evidence that individual differences in communication vary continuously across the population (Campbell and Skarakis-Doyle, 2011; Lancaster and Camarata, 2019) and on dimensional models of autism as a neurodevelopmental condition (Happé and Frith, 2021), the present study aimed to clarify how communication differences are described when accounting for categorical diagnostic group membership and dimensional variation in ASD and ADHD traits across participants. Accordingly, the present study examined how autism and ADHD—considered both categorically and dimensionally—relate to individual differences in communication across ASD, ADHD, and ND groups.

## The present study

In the current study, categorical diagnostic group membership and dimensional trait variation across participants were evaluated to examine how each described differences in communication across ASD, ADHD, and ND groups. The main aim was to investigate whether the relationships between communication abilities and ASD or ADHD traits could be described by a common pattern across the three groups. We tested whether communication scores were more parsimoniously described by dimensional variation in ASD and ADHD traits than by categorical diagnostic group membership alone.

To do this, we considered the Global Communication Composite (GCC) score, which integrates both structural and pragmatic aspects of language. We selected the GCC as the primary outcome because the main aim of the study concerned broad communication difficulties across diagnostic groups, rather than domain-specific profiles. A comprehensive communication index was considered appropriate because both autistic and ADHD participants may show difficulties in structural language (Lord et al., 2004; Andreou et al., 2005; Ellis Weismer et al., 2010; Hudry et al., 2010; Väisänen et al., 2014; Westby and Watson, 2021) and pragmatic skills (Kim and Kaiser, 2000; Norbury, 2005; Bruce et al., 2006; Colle et al., 2008; Staikova et al., 2013; Simmons et al., 2014; Väisänen et al., 2014; Cardillo et al., 2021; Crisci et al., 2022; Çiray et al., 2022). Moreover, using a single global index also limited the risk of overinterpreting domain-specific findings, and reduced the number of primary model comparisons. Exploratory sensitivity analyses for structural and pragmatic composites were also conducted and are reported in the Supplementary materials.

As a central research question, we examined whether communication scores were more parsimoniously described by continuous ASD and ADHD trait measures than by diagnostic group membership. To address this question, we used a model-comparison framework adapted from previous studies comparing dimensional and categorical descriptions in separately recruited clinical and non-diagnosed samples (Mammarella et al., 2021; Carretti et al., 2022). This approach is not a formal taxometric test and does not estimate the latent structure underlying the clinical characteristics of ASD or ADHD. Rather, it compares the extent to which observed communication scores are better described by diagnostic group membership, continuous trait variation, or a combination of both. This logic is conceptually related to comparison-data approaches in taxometric analysis, where observed patterns are evaluated against data generated under alternative categorical and dimensional assumptions (Ruscio et al., 2013; Ruscio et al., 2010). In the present study, however, this logic was applied to the operating characteristics of a predictive model-comparison decision rule and does not provide latent-structure inference.

Because relationships between trait scores and communication outcomes may be non-linear, especially when clinical scales are skewed or affected by diagnostic thresholds, we used Generalized Additive Models (GAMs) (Wood, 2017). Specifically, we compared a set of prespecified candidate models: (a) a categorical model including diagnostic group as the sole predictor, (b) a trait-only model including smooth terms for continuous ASD and ADHD trait measures, and (c) hybrid models combining group membership with trait smoothers, including models with group-specific smoothers.

Based on previous dimensional accounts of neurodevelopmental variation, we expected the trait-only model to provide a parsimonious description of communication outcomes. Specifically, we expected communication skills to be negatively associated with both autistic and ADHD traits across participants. Prior work has shown that higher autistic traits are associated with poorer communication abilities (Demizu et al., 2022), and we expected a similar pattern in the present sample. We also expected ADHD traits to be negatively associated with communication skills across ADHD and non-diagnosed participants (Taylor et al., 2015). Under this framework, finding that the trait-only model fitted at least as well as models including group membership would suggest that communication variation is closely related to continuous ASD and ADHD trait measures in this dataset, while preserving the clinical relevance of diagnostic categories (Caviola et al., 2024; Crisci et al., 2026).

## Method

### Participants and procedure

The study involved 493 participants aged 7 to 16 years, divided into three groups: 147 with ASD without intellectual disability [126 boys; Age = 11.87 (2.92); intelligence quotient (IQ) = 107.12 (14.63)], 105 with ADHD [88 boys; Age = 10.58 (1.96); IQ = 107.26 (11.00)], and 241 ND comparisons [203 boys; Age = 11.37 (2.65); IQ = 110.09 (11.77)].

Inclusion criteria required a standard IQ score of 85 or higher on the Wechsler Intelligence Scales (WISC-IV; Wechsler, 2003). All participants were native Italian speakers with no visual or hearing impairments. Exclusion criteria included current medication use, known genetic conditions, neurological diseases, comorbid psychopathology, and certified physical and intellectual disabilities.

Participants were recruited from clinical centres specialized in ASD and ADHD assessment and intervention, as well as from schools for ND children and adolescents. Participants in the clinical groups were first diagnosed by a multi-professional team including child psychiatrists and psychologists, according to *Diagnostic and Statistical Manual of Mental Disorders* (5th ed.; DSM-5; American Psychiatric Association, 2013) or *International Classification of Diseases* (10th Revision; ICD-10) (World Health Organization, 1992) criteria. The control group consisted of children and adolescents without any psychiatric, neurological, or neurodevelopmental diagnoses.

The study was approved by the Research Ethics Board of the Authors’ institution and adheres to APA ethical standards. After obtaining written parental consent and participants’ verbal assent, certified psychologists tested the participants in sessions lasting about 2 hours. Assessments took place at the referring centers for clinical participants, and at the University for ND families. The sample size was established based on the restricted inclusion and exclusion criteria and the practical constraints related to the availability of clinicians and families for data collection.

### Materials

#### Communication skills

The *Children’s Communication Checklist* (CCC-2, Bishop, 2003; Italian version, Di Sano et al., 2013) is a 70-item caregiver-report questionnaire that evaluates everyday communication skills in children aged 4 to 16 years, focusing on aspects that are challenging to assess with traditional language tests. It includes ten scales: A–D assess structural language (speech, syntax, semantics, coherence); E–H assess pragmatic skills (inappropriate initiation, stereotyped language, use of context, nonverbal communication); I–J assess autistic behaviors. Items are rated from 0 (“less than once a week [or never]”) to 3 (“several times a day [more than twice] or always”), with some reverse scored. Raw scores are converted to scaled scores. In the present study only scales A–H were administered to assess structural and pragmatic communication skills. These scales yield the Global Communication Composite (GCC) score, with lower scores denoting greater communication challenges. A GCC score below 55 suggests potential communication impairments. Cronbach’s alpha = 0.66–0.80 (Bishop, 2003).

#### Autistic symptoms/traits

The *Autism Diagnostic Interview—Revised* (ADI-R; Rutter et al., 2005; Italian version by Fagioli et al., 2005) is a semi-structured parental interview assessing autistic symptoms in individuals aged 2 years old or above. It is composed of 93 items, focusing on three functional domains: reciprocal social interactions, language and communication, and restricted, repetitive, and stereotyped behaviors and interests. The interview questions cover various aspects of the child’s history, behavior, early development, language skills, current abilities, social growth, and other pertinent clinical concerns. Items are scored from 0 (no impairment) to 2 (severe impairment). The scoring algorithms determine a scoring cut-off for each scale. In the present study, the total sum of the three scales was considered in the analyses, with higher scores indicating greater presence of autistic symptoms. Test–retest [ICC = 0.82–0.91; ICC = 0.93–0.97] (Hill et al., 2001; Lord et al., 1993) and inter-rater reliability [ICC = 0.93–0.97; ICC = 0.93–0.97] (Chakrabarti and Fombonne, 2001; Lord et al., 1994) of the ADI-R are very good (Fagioli et al., 2005).

#### ADHD symptoms/traits

The *Conners’ Parent Rating Scale–Revised: Short Form* (CPRS-R: S, Nobile et al., 2007) was employed to assess attention deficits, hyperactivity, and impulsivity traits. It aligns with the criteria outlined in the DSM, including oppositional behaviors commonly observed in ADHD. Administration of the 27-item scale typically required less than 10 min. Parents rated the extent to which these symptoms were characteristic of their child’s behavior over the past month using a 4-point Likert scale ranging from 0 (“not true at all”) to 3 (“very true”). In the present study, the ADHD Index (a set of 12 items) was considered as a global scale. The raw score was converted into a T score. A higher T score indicates a greater presence of ADHD traits. Cronbach’s alpha = 0.75–0.94 (Conners et al., 1998).

### Analytical strategy

Data analysis was conducted using the free R software (R Core Team, 2024). Generalized Additive Models (GAMs) were fitted using the “mgcv” package of R (Wood, 2017).

Descriptive statistics (M, SD, skewness, kurtosis) were computed as a preliminary analysis for communication ability scores (GCC), ASD clinical scale (ADI-R) scores, and ADHD index (CPRS-R: S). Departures from normality were assessed using the Shapiro–Wilk test. When conducting categorical analysis, homogeneity of variances was tested using Levene’s test, and variance ratios were calculated to estimate differences in variability across groups. Standardized mean differences (Cohen’s 𝑑) were then computed to compare group means. Particular attention was given to how each clinical group (ADHD and ASD) differed from the ND on GCC.

We then used a model-comparison approach to examine whether GCC scores were more parsimoniously described by diagnostic group membership, continuous ASD and ADHD trait scores, or both. This analysis was not intended as a formal taxometric test and does not assess whether ASD or ADHD clinical diagnoses are latent categories or dimensions. It evaluates which set of observed predictors provides the most parsimonious description of communication scores in this sample.

As a preliminary descriptive step, we computed bivariate correlations between GCC and each trait measure within the ND group. These correlations provide a simple benchmark for the association between communication scores and trait variation in participants without a diagnosis. Because the ND group has a restricted range of trait scores by design, these correlations were interpreted cautiously.

We then fitted Generalized Additive Models (GAMs), which allow the association between trait scores and GCC to be non-linear when needed (Wood, 2017). We compared prespecified candidate models: (a) a categorical model including diagnostic group as the only predictor, (b) a trait-only model including smooth terms for ADHD and ASD trait scores, and (c) hybrid models combining diagnostic group and trait smoothers, including models with group-specific smoothers. In intuitive terms, if the trait-only model fits better than the group-only model, this means that communication scores are described more parsimoniously by continuous trait variation than by diagnostic group membership alone. If adding group membership improves fit after accounting for trait scores, this indicates that diagnostic group still contributes information beyond the continuous trait measures.

Model comparison was based on the Bayesian Information Criterion (BIC), with lower values indicating a better balance between fit and complexity. BIC was used because it provides a fit-to-parsimony criterion for comparing prespecified candidate descriptions of the same outcome (Aho et al., 2014). We used it as a model-selection criterion, with no implication that it provides a stand-alone test of dimensionality. Following recommendations for model-comparison frameworks, we paired this criterion with a simulation-based model-recovery study to examine whether the decision rule could distinguish among the candidate accounts under conditions resembling the empirical sample (Palminteri et al., 2017; Wilson and Collins, 2019). Overfitting was also evaluated through standard GAM diagnostics, including effective degrees of freedom, basis-dimension checks, visual inspection of the fitted smooths, and concurvity diagnostics. Because group membership and trait scores were strongly related by design, hybrid models were interpreted cautiously.

Predicted effects from the final model were plotted for descriptive purposes to illustrate the fitted trait-GCC associations within the observed score ranges. These plots were used to support interpretation of the model-comparison results, not as independent evidence about the latent structure of ASD or ADHD.

Full diagnostics and additional checks are reported in the Supplementary materials. Specifically, Supplementary materials include: additional GAM plots with Predicted GCC values from the flexible hybrid GAM, plotted separately for ND, ADHD, and ASD groups; measurement checks for the GCC and communication subcomponents; additional GAM model comparisons and sensitivity analyses (to evaluate whether the main pattern was robust to covariate adjustment, alternative outcome definitions, and reduced overlap between the ADI-R predictor and GCC outcome); and a set of simulations to check the plausibility of the modelling strategy (not as formal validation).

Data and codes are available via the OSF repository: https://osf.io/u7r4t/overview?view_only=a74a3595d44749799a44b3072dc65641. We report how we determined our sample size, all manipulations, and all measures in the study. However, the materials used in this study cannot be shared publicly as they are clinical instruments protected by copyright and commercial distribution rights.

## Results

Table 1 presents descriptive statistics for the GCC, ADHD index, and ADI-R score across the three groups. The Shapiro–Wilk test revealed significant departures from normality in most cases.

**Table 1:** Descriptive statistics and test of normality for the three main variables of interest in the three groups [Non-Diagnosed (ND), *n* = 241; ADHD, *n* = 105; ASD, *n* = 147].

Group	M (SD)	Skewness	Kurtosis	Shapiro–Wilk test	
				W	*p*
Variable: GCC score (sum of weighted scores; population average = 80)					
ND	77.98 (19.63)	−0.34	−0.42	0.97	**< 0.001**
ADHD	58.05 (20.86)	−0.06	−0.81	0.98	0.131
ASD	46.73 (17.52)	0.66	0.40	0.97	**0.002**
Variable: ADHD index (T scores; population average = 50)					
ND	48.79 (8.65)	1.15	0.91	0.89	**< 0.001**
ADHD	74.64 (9.97)	0.40	0.60	0.96	**0.007**
ASD	63.51 (13.23)	0.21	−0.82	0.98	**0.009**
Variable: ADI-R (sum of raw scores; range [0, 63])					
ND	7.46 (5.83)	1.60	3.28	0.87	**< 0.001**
ADHD	13.41 (8.02)	0.71	0.11	0.95	**0.001**
ASD	33.31 (11.70)	−0.06	−0.27	0.99	0.686

GCC score, Global communication composite score; ADHD index, ADHD symptoms/traits; ADI-R, autism symptoms/traits. For perfect normality, skewness = 0, kurtosis = 0. Bold values indicate statistically significant results of the Shapiro-Wilk test.

As for categorical comparison, although the GCC scores appeared to have similar standard deviations across the three groups, Levene’s test for homogeneity of variance indicated heteroscedasticity, *F* (2, 490) = 3.57, *p* = 0.03. Variance ratios were 1.13 for ADHD vs. ND and 0.80 for ASD vs. ND. These departures from normality and heteroscedasticity make the interpretation of standardized mean differences (SMDs) potentially problematic, as extreme values may disproportionately influence both the mean and standard deviation, and the pooled SD may not accurately represent the variability within either group.

Nevertheless, we proceeded with SMDs cautiously, primarily for descriptive purposes, noting that in the case of GCC, neither the departures from normality nor the heteroscedasticity were quantitatively extreme. When comparing the ADHD group with the ND group on GCC, *Cohen’s d* = −1.00 [95% CI: −1.24, −0.75]. When comparing the ASD group with the ND group on GCC, *d* = −1.67 [95% CI: −1.90, −1.43]. Both SMDs were very large, as expected, indicating substantial difficulties in communication abilities in both clinical groups. Consistent with expectations, the ASD-ND difference was descriptively larger than the ADHD-ND difference. A formal group comparison supported this pattern: the overall group effect was significant, *F* (2, 490) = 127.09, *p* < 0.001, and post-hoc contrasts indicated that the ASD group had lower GCC scores than the ADHD group, *mean difference* = −11.3, *SE* = 2.43, *p* < 0.001. A bootstrap test on the difference between standardized effect sizes (10,000 replications) confirmed this asymmetry: *d*(ASD − ND) − *d*(ADHD−ND) = −0.67, 95% CI [−1.05, −0.31]. In examining dimensionality, as an initial step, we computed simple bivariate correlations between GCC and each clinical scale within the ND group. GCC showed negative correlations with the ADHD index (*r* = −0.41) and the ADI-R (*r* = −0.53). When these correlation coefficients were converted to *Cohen’s d*, the resulting values were *d* = −0.91 and *d* = −1.27, respectively.

As a second step, we compared the prespecified GAM models using the Bayesian Information Criterion (BIC; lower values indicating better balance of fit and complexity).

We compared three main model families: a categorical model including diagnostic group only, a trait-based model including smooth terms for ADHD and ASD traits, and hybrid models combining group and trait predictors. The categorical model showed substantially poorer fit (BIC = 4324.75) than models including continuous trait scores. The lowest BIC was obtained by the trait-only GAM, including smooth terms for ADHD and ASD traits and no group predictor (BIC = 4151.39). This model also outperformed the best hybrid alternative (BIC = 4159.74; ΔBIC = 8.35). Thus, within this model-selection framework, adding diagnostic group did not improve the description of GCC scores once continuous trait scores were included. In the final model, the ADHD smoother used 2.04 effective degrees of freedom, and the ADI-R smoother used 3.33 effective degrees of freedom, indicating limited non-linearity.

This result should be interpreted as evidence that continuous trait scores captured communication variation more parsimoniously than diagnostic labels in this dataset. It should not be interpreted as evidence about the latent structure of ASD or ADHD. Full model comparisons, concurvity diagnostics, and sensitivity checks are reported in the Supplementary materials.

**Figure 1:**
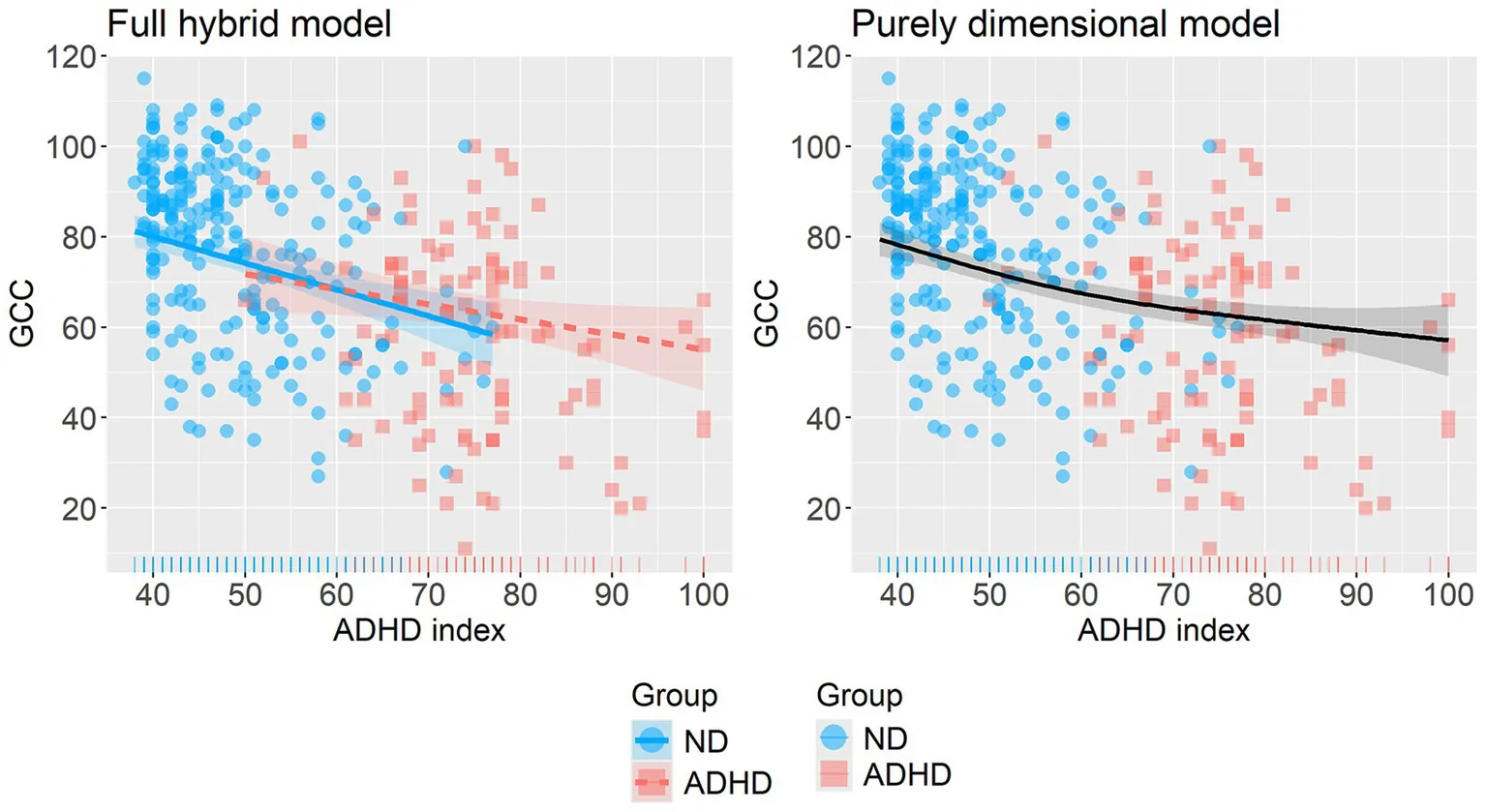
Predicted marginal effects of the ADHD index on GCC from the full hybrid and final trait-only GAM models for the non-diagnosed (ND) and ADHD groups. GCC, Global communication composite score; ADHD index, ADHD symptoms/traits. Rug marks show the observed distribution of ADHD index scores by group. The trait-only model estimates a common smooth function across the whole sample, whereas the full hybrid model allows group-specific smooth functions. Regions with sparse observations should be interpreted cautiously.

**Figure 2:**
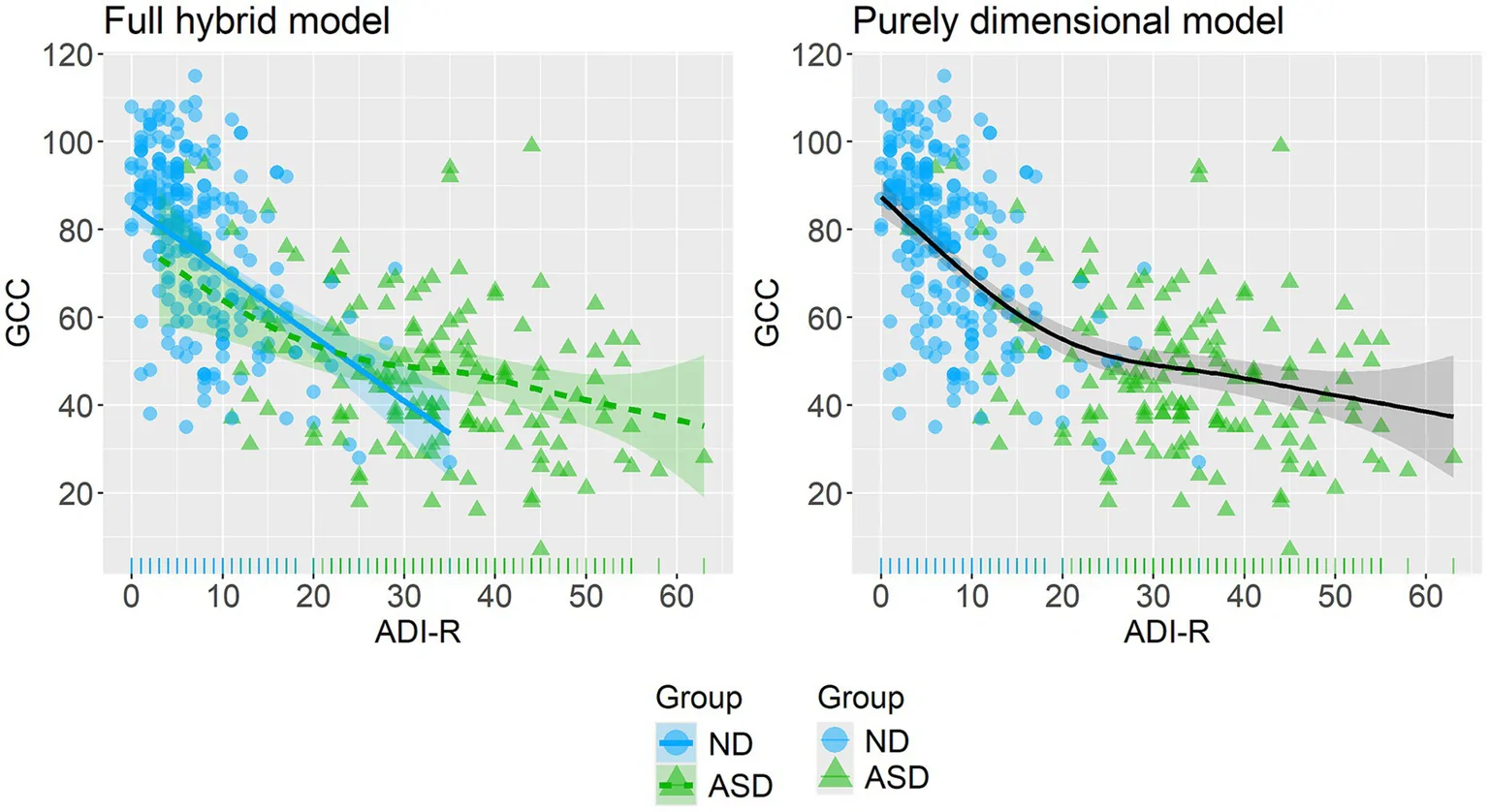
Predicted marginal effects of ADI-R on GCC from the full hybrid and final trait-only GAM models for the non-diagnosed (ND) and ASD groups. GCC, global communication composite score; ADI-R, autism symptoms/traits. Rug marks show the observed distribution of ADI-R scores by group. The trait-only model estimates a common smooth function across the whole sample, whereas the full hybrid model allows group-specific smooth functions. Regions with sparse observations should be interpreted cautiously.

**Figure 3:**
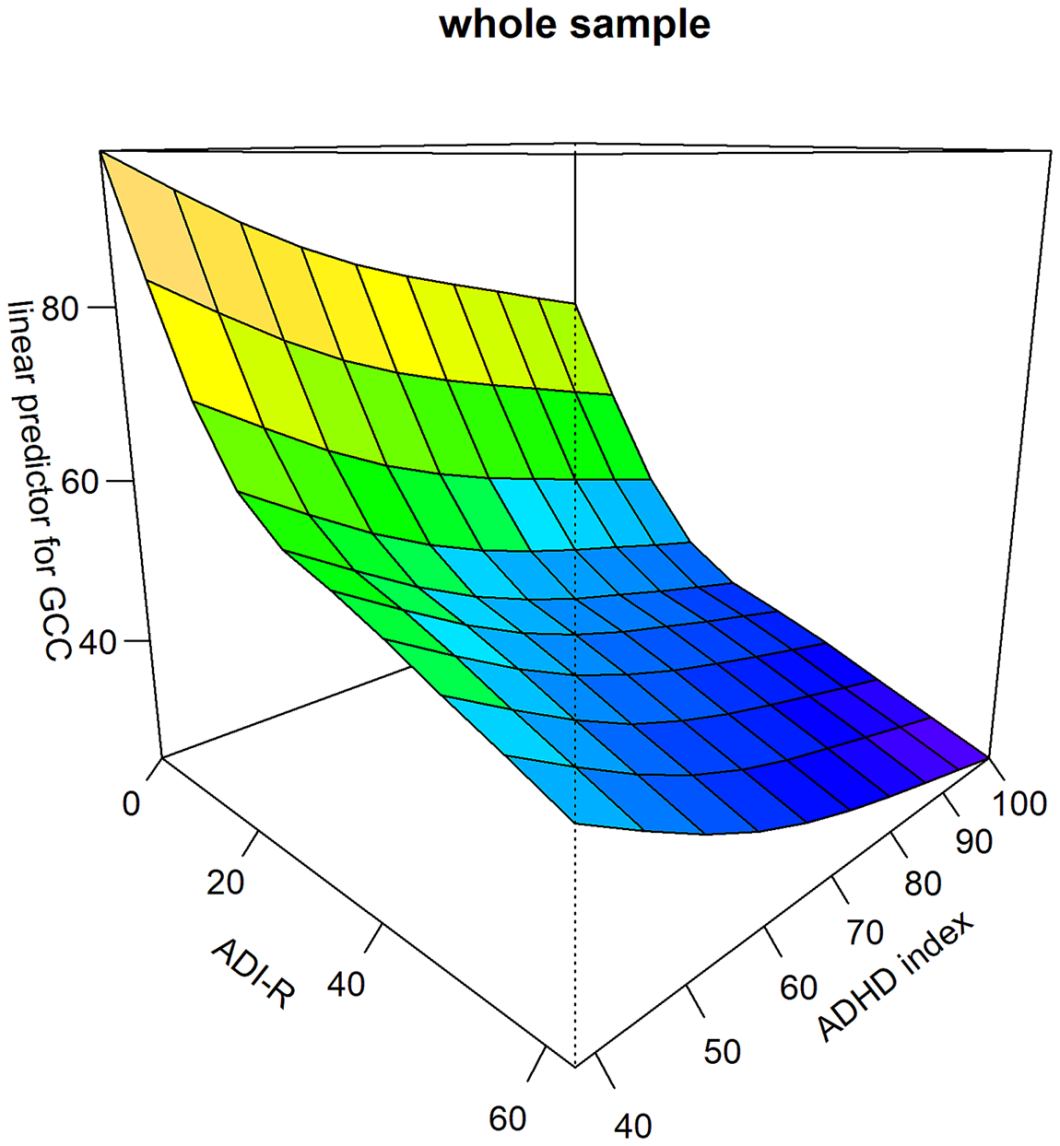
3D plot showing the predicted GCC scores from the final trait-only GAM model, which includes smooth terms for both ADHD index and ADI-R and no group predictor. The surface shows the joint association of ADHD and autistic traits with GCC scores, with modest non-linearity. GCC, Global communication composite score; ADHD index, ADHD symptoms/traits; ADI-R, autism symptoms/traits. The figure is descriptive and should be interpreted with caution in regions with few observations.

Figures 1, 2 compare predictions from the full hybrid model and the final trait-only model, separately for ADHD traits and autistic traits. Rug marks indicate the observed distribution of trait scores by group, helping to identify the regions of the smooth functions that are well supported by observed data. The full hybrid model allows group-specific flexibility, whereas the trait-only model estimates a common function across the whole sample. The two approaches produced similar functional forms within the trait-score ranges shared across groups, while the trait-only model provided the more parsimonious fit. Regions with few observations, especially at the extremes of the trait distributions, should be interpreted cautiously. Figure 3 provides a descriptive 3D representation of the predicted GCC surface from the final trait-only model, showing the joint association of ADHD and autistic traits with GCC scores. To assess whether a simpler linear approach would suffice, we additionally fitted standard linear models without smoothing terms. The best such model (BIC = 4181.56) performed substantially worse than the dimensional GAM (BIC = 4151.39), indicating that allowing limited non-linearity provided a more adequate representation of the observed patterns.

Finally, following recommendations to pair model comparison with generative model-recovery checks (Palminteri et al., 2017; Wilson and Collins, 2019), we conducted a simulation-based evaluation of the GAM-BIC decision rule. The aim was to examine whether the same procedure could recover the dominant data-generating pattern under conditions resembling the empirical sample, including similar group sizes, trait distributions, scale truncation, and modest non-linearity. We simulated three scenarios: a trait-driven scenario, a group-driven scenario, and a hybrid scenario combining both sources of information. Across 1,000 replications per scenario, the procedure selected the expected model in 98.8% of trait-driven datasets, 99.8% of group-driven datasets, and 87.0% of hybrid datasets. When requiring stronger evidence, defined as ΔBIC > 6 between the best and second-best models, recovery rates were 91.8, 98.2, and 65.3%, respectively. These simulations establish the operating characteristics of the decision rule under empirically calibrated scenarios. They do not validate the latent structure underlying the clinical characteristics of ASD or ADHD, and the inference they support remains conditional on the realism of the simulated scenarios. Full details are reported in the Supplementary materials.

## Discussion

This study examined communication skills in children and adolescents with ASD, ADHD, and non-diagnosed (ND) peers. Building on evidence that communication abilities vary continuously across the population, the present study examined whether differences in communication are better described by categorical diagnostic group membership or by dimensional variation in ASD and ADHD traits across individuals. In other words, using Generalized Additive Models, the study compared whether communication scores were more parsimoniously described by diagnostic group membership, continuous ASD/ADHD trait scores, or both.

Preliminary analyses revealed that parents of children in both clinical groups reported lower GCC scores compared to those of ND participants (Helland et al., 2012; Roselló et al., 2017; Mammarella et al., 2022). However, the effect size of the comparison with ND peers was significantly larger for the ASD group than that for the ADHD group. While the mean GCC score for the ASD group fell below the clinical threshold (<55), the mean score for the ADHD group was slightly higher, indicating milder, but still notable, communication difficulties that may impact daily functioning and warrant further assessment. This pattern aligns with previous studies showing that autistic individuals often present with greater communication impairments than non-autistic peers, including those with ADHD (Ghaziuddin et al., 2010; Mouti et al., 2019). In particular, individuals with ADHD tend to perform better than autistic individuals on measures of stereotyped language and non-verbal communication (Roselló et al., 2017). Nonetheless, children with ADHD appear to face greater communication challenges than their ND peers (Kochhar et al., 2011; Mouti et al., 2019). While some studies report distinct communication profiles in ASD and ADHD (Salley et al., 2015), others highlight overlap with children with ADHD displaying language patterns similar to those in ASD (Geurts and Embrechts, 2008; Mammarella et al., 2022). Clinically, communication difficulties are commonly associated with the core characteristics of ASD and, to a lesser extent, ADHD, but they are not confined to individuals with a formal clinical diagnosis. While categorical approaches are essential for identifying clinically significant impairments, they may be less well suited to capturing the extent to which subclinical ASD and ADHD traits are also associated with variation in communication abilities. This highlights the value of complementing categorical classifications with a dimensional perspective, as explored in the central part of our study.

To test our main aim, we analysed the covariation between ASD and ADHD traits and communication across groups. This dimensional approach allows us to capture a broader range of individual differences, revealing shared characteristics and underlying mechanisms that may be overlooked in traditional categorical frameworks (Roefs et al., 2022; Caviola et al., 2024). Our results suggest that communication challenges vary systematically in relation to both autistic (Landa et al., 1992; Ingersoll, 2010; Reindal et al., 2023) and ADHD traits (Nilsen et al., 2015; Rints et al., 2015; Hawkins et al., 2016; Parks et al., 2021) across the three groups, including participants without a clinical diagnosis. Model comparisons showed that the final models excluding the group variable performed significantly better than those including it. It is important to note, however, that superior model fit in terms of predictive performance does not determine the latent structure of ASD or ADHD as clinical constructs. Rather, our findings indicate that the dimensional model provided a better account of communication scores within our dataset and should not be interpreted as resolving the broader question of whether ASD and ADHD are best conceptualized as categorical or dimensional conditions. Additionally, from a clinical standpoint, our results do not diminish the value of formal diagnoses. Rather, they emphasize that both diagnosed and ND children may experience varying degrees of communication and language difficulties that are consistent with quantitative differences in ASD and ADHD traits, highlighting the need for tailored support. Formal diagnoses remain essential for guiding access to services, informing intervention planning, and capturing the broader constellation of strengths and difficulties that define neurodevelopmental conditions. Rather than competing with a categorical diagnostic framework, the dimensional signal observed here should be viewed as complementary, highlighting that communication difficulties exist on a continuum that extends beyond formal diagnostic boundaries. Thus, such difficulties may also be present to varying degrees, in children who do not meet full diagnostic criteria, but nonetheless may benefit from appropriate support (see also Crisci et al., 2026). Interestingly, lower ADI-R scores (i.e., less severe autism-related symptoms) were associated with higher GCC scores, but this negative relationship diminished as ASD traits increased, likely due to the plateauing effect in GCC scores. In contrast, the association between GCC and the ADHD index appeared weaker than that observed for ASD traits in the general population, as reflected in both the bivariate correlations and effect sizes. These findings reinforce the results from the categorical analysis, suggesting that while autistic traits show a consistent relationship with communication difficulties (Mouti et al., 2019; Cardillo et al., 2021; Demizu et al., 2022; Reindal et al., 2023), ADHD traits may have a more indirect effect, possibly mediated—as previously suggested—by factors like impulsivity (Baird et al., 2000; Parks et al., 2021) and executive function deficits (Hawkins et al., 2016).

Our findings may have important clinical implications for the assessment and treatment of communication difficulties in children and adolescents. On one hand, a dimensional framework would help identify communication challenges in individuals who do not meet diagnostic criteria, enabling more personalized interventions. On the other hand, as previously mentioned, formal diagnoses remain essential in clinical contexts to ensure access to appropriate support and services. As our findings suggest, children—regardless of diagnostic status—may exhibit varying degrees of communication impairment, ranging from mild to severe. This perspective encourages practitioners to adopt more individualized strategies. In summary, adopting a dimensional approach to assessment and intervention may enable clinicians to more effectively address the diverse communication needs of both diagnosed and ND youth.

This study offers innovative contributions to the investigation of communication skills and neurodevelopment variation, while also acknowledging limitations that can inform future research. From a methodological standpoint, clinical tools for assessing ASD and ADHD are designed to capture symptoms at a diagnostic threshold, often leading to skewed score distributions. The observed non-linearity in our results was characterized by a steeper curve at the lower end of the clinical scales, which aligns with expectations due to the restricted range of scores within the ND group. Indeed, diagnostic tools may not fully capture the distribution of traits in the general population. When the aim is not just to identify significant deficits in narrowly defined groups but to evaluate dimensions across all levels of functioning, further research should devise tools that effectively measure traits along the entire continuum (Caviola et al., 2024). Related to this, a further conceptual limitation concerns the nature of the dimensional predictors used in our models. The continuous trait measures employed in this study derive from instruments originally developed within categorical diagnostic frameworks. As such, the dimensional model is not fully independent from the categorical structure it is compared against. This circularity limits the extent to which our findings can be interpreted. More specifically, when categorical instruments are repurposed to derive continuous scores, the resulting measures may retain implicit categorical assumptions embedded in their construction. Future research would benefit from employing measures explicitly designed to capture trait variability across both clinical and non-clinical populations—such as broadband dimensional scales—which would reduce circularity and provide a more neutral and ecologically valid test of the categorical versus dimensional debate. A further methodological limitation concerns the potential for criterion contamination between the ADI-R total score and the CCC-2 GCC. Because the ADI-R includes communication-related content, part of the association between autistic traits and GCC may reflect overlapping measurement content. However, as suggested by our analyses, the main finding was not fully driven by communication-content overlap. At the same time, excluding communication-related content from the ADI-R yields a narrower index of autistic traits, and the sensitivity analysis does not remove the broader limitation that all measures were based on related behavioral ratings. Future studies should use more construct-independent measures of autistic traits and communication outcomes. Furthermore, our study focused solely on communication, overlooking other factors commonly present in both ASD and ADHD. Future research should extend this approach to other shared areas of difficulties, adopting a dimensional framework to better capture the complexity and heterogeneity of neurodevelopmental profiles. Another key limitation of our study is the reliance on a single informant, which introduces a risk of shared-method variance. This limitation is especially relevant for the present model comparison. Because all measures were caregiver-reported, response style, rater consistency, and implicit theories about the child may have inflated the covariance among continuous trait and communication measures, potentially favoring trait-based models over the group-only model in BIC-based comparisons. The advantage of the trait-only model should therefore be interpreted as applying to caregiver-reported communication outcomes, and not as evidence of construct-level continuity. It should be noted, however, that the reliance on caregiver report was methodologically deliberate: research on neurodevelopmental conditions has consistently documented substantial inter-informant discrepancies (Lerner et al., 2012; Wanstall et al., 2019), particularly for concrete behavioral dimensions, and caregiver report was considered the most ecologically valid and consistent source of information across participants (Kenworthy et al., 2022; Shipkova et al., 2025). Future research should include multi-informant reports and direct observations to provide a more comprehensive evaluation. Furthermore, the stringent exclusion criteria—including intellectual disability, comorbid psychopathology, and medication use—produced a highly homogeneous sample that may not be representative of clinical populations, where comorbidity is the rule rather than the exception. These exclusions were intentional, aimed at isolating the specific contribution of autistic and ADHD traits to communication outcomes while minimizing confounding variance introduced by co-occurring conditions. Nevertheless, the dimensional pattern observed here may not generalize to more heterogeneous samples and across varying levels of severity, and replication in clinically representative populations is warranted. Finally, the wide age range (7–16 years) reflects the practical constraints of recruitment, as participation depended on the availability of families and clinical services. While age was included as a covariate in additional analyses and did not substantially alter the findings, developmental variation across this window should be considered when interpreting results. Similarly, the sample was heavily male-skewed, consistent with the known epidemiology of ASD and ADHD (Bölte et al., 2023) but limiting the generalizability of findings. Future research should specifically include an equal sex distribution in clinical groups but also examine whether the dimensional trait-communication associations observed here hold across sexes. Research has consistently shown that autistic girls often display distinct communicative profiles compared to their male counterparts, partly due to camouflaging strategies—such as masking autistic traits through deliberate imitation of social behavior and suppression of difficulties (Hull et al., 2020). This phenomenon could attenuate the observed associations between autistic traits and communication outcomes in females, and might also influence the relative fit of dimensional versus categorical models.

To conclude, this study shows that communication difficulties in this sample were systematically associated with continuous ASD and ADHD trait measures across children and adolescents with and without clinical diagnoses. Across both categorical and dimensional analyses, autistic traits showed a strong and consistent association with communication impairments, whereas ADHD traits exhibited a weaker but still significant association. These findings support the usefulness of considering continuous neurodevelopmental trait measures when studying communication challenges, alongside formal diagnostic categories.

## Data Availability

The datasets presented in this study can be found in online repositories. The names of the repository/repositories and accession number(s) can be found in the article/Supplementary material.

## References

[ref1] Aho K. Derryberry D. Peterson T. (2014). Model selection for ecologists: the worldviews of AIC and BIC. Ecology 95, 631–636. doi: 10.1890/13-1452.1, 24804445

[ref2] American Psychiatric Association (2013). Diagnostic and Statistical Manual of Mental Disorders. Washington: American Psychiatric Association.

[ref3] Andreou G. Agapitou P. Karapetsas A. (2005). Verbal skills in children with ADHD. Eur. J. Spec. Needs Educ. 20, 231–238. doi: 10.1080/08856250500055743

[ref4] Andrés-Roqueta C. Katsos N. (2017). The contribution of grammar, vocabulary and theory of mind in pragmatic language competence in children with autistic Spectrum disorders. Front. Psychol. 8:996. doi: 10.3389/fpsyg.2017.00996, 28663734 PMC5471327

[ref5] Baird J. Stevenson J. C. Williams D. C. (2000). The evolution of ADHD: a disorder of communication? Q. Rev. Biol. 75, 17–35. doi: 10.1086/393256, 10721532

[ref6] Bellani M. Moretti A. Perlini C. Brambilla P. (2011). Language disturbances in ADHD. Epidemiol. Psychiatr. Sci. 20, 311–315. doi: 10.1017/S2045796011000527, 22201208

[ref7] Bishop D. V. (2003). The Children's Communication Checklist. Agra: Psychological Corporation.

[ref8] Bishop D. V. Baird G. (2001). Parent and teacher report of pragmatic aspects of communication: use of the children’s communication checklist in a clinical setting. Dev. Med. Child Neurol. 43, 809–818. doi: 10.1017/s0012162201001475, 11769267

[ref9] Bölte S. Neufeld J. Marschik P. B. Williams Z. J. Gallagher L. Lai M.-C. (2023). Sex and gender in neurodevelopmental conditions. Nat. Rev. Neurol. 19, 136–159. doi: 10.1038/s41582-023-00774-6, 36747038 PMC10154737

[ref10] Bruce B. Thernlund G. Nettelbladt U. (2006). ADHD and language impairment. Eur. Child Adolesc. Psychiatry 15, 52–60. doi: 10.1007/s00787-006-0508-9, 16514510

[ref11] Camarata S. M. Gibson T. (1999). Pragmatic language deficits in attention-deficit hyperactivity disorder (ADHD). Ment. Retard. Dev. Disabil. Res. Rev. 5, 207–214. doi: 10.1002/(SICI)1098-2779(1999)5:33C207::AID-MRDD73E3.0.CO;2-O

[ref12] Campbell W. N. Skarakis-Doyle E. (2011). The relationship between peer conflict resolution knowledge and peer victimization in school-age children across the language continuum. J. Commun. Disord. 44, 345–358. doi: 10.1016/j.jcomdis.2011.01.005, 21367428

[ref13] Cardillo R. Mammarella I. C. Demurie E. Giofrè D. Roeyers H. (2021). Pragmatic language in children and adolescents with autism Spectrum disorder: do theory of mind and executive functions have a mediating role? Autism Res. 14, 932–945. doi: 10.1002/aur.2423, 33111475

[ref14] Carretti B. Cornoldi C. Antonello A. Di Criscienzo L. Toffalini E. (2022). Inferring the performance of children with dyslexia from that of the general population: the case of associative phonological working memory. Sci. Stud. Read. 26, 47–60. doi: 10.1080/10888438.2021.1897596

[ref15] Carruthers S. Taylor L. Sadiq H. Tripp G. (2022). The profile of pragmatic language impairments in children with ADHD: a systematic review. Dev. Psychopathol. 34, 1938–1960. doi: 10.1017/S0954579421000328, 33973504

[ref16] Caviola S. Greiff S. Toffalini E. (2024). Learning disorders and difficulties: from a categorical to a dimensional perspective. Learn. Individ. Differ. 113:102490. doi: 10.1016/j.lindif.2024.102490

[ref17] Chakrabarti S. Fombonne E. (2001). Pervasive developmental disorders in preschool children. JAMA 285, 3093–3099. doi: 10.1001/jama.285.24.3093, 11427137

[ref18] Chown N. Leatherland J. (2021). Can a person be ‘a bit autistic’? A response to Francesca Happé and Uta Frith. J. Autism Dev. Disord. 51, 749–751. doi: 10.1007/s10803-020-04541-0, 32535669

[ref19] Çiray R. O. Özyurt G. Turan S. Karagöz E. Ermiş Ç. Öztürk Y. . (2022). The association between pragmatic language impairment, social cognition and emotion regulation skills in adolescents with ADHD. Nord. J. Psychiatry 76, 89–95. doi: 10.1080/08039488.2021.1938211, 34182872

[ref20] Colle L. Baron-Cohen S. Wheelwright S. van der Lely H. K. J. (2008). Narrative discourse in adults with high-functioning autism or Asperger syndrome. J. Autism Dev. Disord. 38, 28–40. doi: 10.1007/s10803-007-0357-5, 17345168

[ref21] Conners C. K. Sitarenios G. Parker J. D. A. Epstein J. N. (1998). The revised Conners’ parent rating scale (CPRS-R): factor structure, reliability, and criterion validity. J. Abnorm. Child Psychol. 26, 257–268. doi: 10.1023/A:1022602400621, 9700518

[ref22] Crisci G. Cardillo R. Mammarella I. C. (2022). The processes underlying positive illusory Bias in ADHD: the role of executive functions and pragmatic language skills. J. Atten. Disord. 26, 1245–1256. doi: 10.1177/10870547211063646, 34937413

[ref23] Crisci G. Lievore R. Mammarella I. C. (2026). Blurred lines: investigating functional profiles in autism and ADHD across key developmental domains. Res. Dev. Disabil. 170:105240. doi: 10.1016/j.ridd.2026.105240, 41724026

[ref24] de la Torre Carril A. Durán-Bouza M. Pérez-Pereira M. (2021). Capacity of the CCC-2 to discriminate ASD from other neurodevelopmental disorders. Children 8:640. doi: 10.3390/children8080640, 34438530 PMC8391826

[ref25] Demizu Y. Matsumoto J. Yasuda Y. Ito S. Miura K. Yamamori H. . (2022). Relationship between autistic traits and social functioning in healthy individuals. Neuropsychopharmacol. Rep. 42, 226–229. doi: 10.1002/npr2.12249, 35365959 PMC9216356

[ref26] Di Sano S. Saggino A. Barbieri M. S. Luca S. (2013). “Adattamento italiano della Children's Communication Checklist 2,” in Children's Communication Checklist-Second Edition, (Florence: Giunti O.S).

[ref27] Dolata J. K. Suarez S. Calamé B. Fombonne E. (2022). Pragmatic language markers of autism diagnosis and severity. Res. Autism Spectr. Disord. 94:101970. doi: 10.1016/j.rasd.2022.101970, 35528460 PMC9075340

[ref28] Ellis Weismer S. Lord C. Esler A. (2010). Early language patterns of toddlers on the autism Spectrum compared to toddlers with developmental delay. J. Autism Dev. Disord. 40, 1259–1273. doi: 10.1007/s10803-010-0983-1, 20195735 PMC2941531

[ref29] Fagioli R. Saccani M. Persico A. M. Tancredi R. Parrini B. Igliozzi R. (2005). Autism Diagnostic Interview-Revised. Italian Version. Florence: Giunti O.S.

[ref30] Ferrara M. Camia M. Cecere V. Villata V. Vivenzio N. Scorza M. . (2020). Language and pragmatics across neurodevelopmental disorders: an investigation using the Italian version of CCC-2. J. Autism Dev. Disord. 50, 1295–1309. doi: 10.1007/s10803-019-04358-6, 31901121

[ref31] Geurts H. M. Embrechts M. (2008). Language profiles in ASD, SLI, and ADHD. J. Autism Dev. Disord. 38, 1931–1943. doi: 10.1007/s10803-008-0587-1, 18521730

[ref32] Geurts H. M. Verté S. Oosterlaan J. Roeyers H. Hartman C. A. Mulder E. J. . (2004). Can the children’s communication checklist differentiate between children with autism, children with ADHD, and normal controls? J. Child Psychol. Psychiatry 45, 1437–1453. doi: 10.1111/j.1469-7610.2004.00326.x, 15482504

[ref33] Ghaziuddin M. Welch K. Mohiuddin S. Lagrou R. Ghaziuddin N. (2010). Utility of the social and communication questionnaire in the differentiation of autism from ADHD. J. Dev. Phys. Disabil. 22, 359–366. doi: 10.1007/s10882-010-9199-8

[ref34] Green B. C. Johnson K. A. Bretherton L. (2014). Pragmatic language difficulties in children with hyperactivity and attention problems: an integrated review. Int. J. Lang. Commun. Disord. 49, 15–29. doi: 10.1111/1460-6984.12056, 24372883

[ref35] Grove W. M. (1991). “Validity of taxometric inferences based on cluster analysis stopping rules,” in Thinking Clearly about Psychology: Essays in honor of Paul E. Meehl, Vol. 1: Matters of public Interest; Vol. 2: Personality and Psychopathology, (Minneapolis: University of Minnesota Press).

[ref36] Happé F. Frith U. (2020). Annual research review: looking back to look forward – changes in the concept of autism and implications for future research. J. Child Psychol. Psychiatry 61, 218–232. doi: 10.1111/jcpp.13176, 31994188

[ref37] Happé F. Frith U. (2021). Dimensional or categorical approaches to autism? Both are needed. a reply to Nick Chown and Julia Leatherland. J. Autism Dev. Disord. 51, 752–753. doi: 10.1007/s10803-020-04728-5, 33006107

[ref38] Hawkins E. Gathercole S. Astle D. The CALM Team Holmes J. (2016). Language problems and ADHD symptoms: how specific are the links? Brain Sci. 6:50. doi: 10.3390/brainsci6040050, 27775648 PMC5187564

[ref39] Helland W. A. Biringer E. Helland T. Heimann M. (2012). Exploring language profiles for children with ADHD and children with Asperger syndrome. J. Atten. Disord. 16, 34–43. doi: 10.1177/1087054710378233, 20837976

[ref40] Helland W. A. Posserud M.-B. Lundervold A. J. (2022). Emotional and behavioral function in children with language problems a longitudinal, population based study. Eur. J. Spec. Needs Educ. 37, 177–190. doi: 10.1080/08856257.2020.1857930

[ref41] Hill A. Bölte S. Petrova G. Beltcheva D. Tacheva S. Poustka F. (2001). Stability and interpersonal agreement of the interview-based diagnosis of autism. Psychopathology 34, 187–191. doi: 10.1159/000049305, 11549928

[ref42] Hudry K. Leadbitter K. Temple K. Slonims V. McConachie H. Aldred C. . (2010). Preschoolers with autism show greater impairment in receptive compared with expressive language abilities. Int. J. Lang. Commun. Disord. 45, 681–690. doi: 10.3109/13682820903461493, 20102259

[ref43] Hull L. Petrides K. V. Mandy W. (2020). The female autism phenotype and camouflaging: a narrative review. Rev. J. Autism Dev. Disord. 7, 306–317. doi: 10.1007/s40489-020-00197-9

[ref44] Ingersoll B. (2010). Broader autism phenotype and nonverbal sensitivity: evidence for an Association in the General Population. J. Autism Dev. Disord. 40, 590–598. doi: 10.1007/s10803-009-0907-0, 19936903

[ref45] Jepsen I. B. Brynskov C. Thomsen P. H. Rask C. U. Jensen de López K. Lambek R. (2024). The role of language in the social and academic functioning of children with ADHD. J. Atten. Disord. 28, 1542–1554. doi: 10.1177/10870547241266419, 39077785

[ref46] Kenworthy L. Verbalis A. Bascom J. daVanport S. Strang J. F. Pugliese C. . (2022). Adding the missing voice: how self-report of autistic youth self-report on an executive functioning rating scale compares to parent report and that of youth with attention deficit hyperactivity disorder or neurotypical development. Autism 26, 422–433. doi: 10.1177/13623613211029117, 34238038 PMC8742839

[ref47] Kessler P. B. Ikuta T. (2023). Pragmatic deficits in attention deficit/hyperactivity disorder: systematic review and Meta-analysis. J. Atten. Disord. 27, 812–821. doi: 10.1177/10870547231161534, 36975307

[ref48] Kim O. H. Kaiser A. P. (2000). Language characteristics of children with ADHD. Commun. Disord. Q. 21, 154–165. doi: 10.1177/152574010002100304

[ref49] Kochhar P. Batty M. J. Liddle E. B. Groom M. J. Scerif G. Liddle P. F. . (2011). Autistic spectrum disorder traits in children with attention deficit hyperactivity disorder. Child Care Health Dev. 37, 103–110. doi: 10.1111/j.1365-2214.2010.01123.x, 20666783

[ref50] Korrel H. Mueller K. L. Silk T. Anderson V. Sciberras E. (2017). Research review: language problems in children with attention-deficit hyperactivity disorder—a systematic meta-analytic review. J. Child Psychol. Psychiatry 58, 640–654. doi: 10.1111/jcpp.12688, 28186338

[ref51] Kotov R. Krueger R. F. Watson D. Achenbach T. M. Althoff R. R. Bagby R. M. . (2017). The hierarchical taxonomy of psychopathology (HiTOP): a dimensional alternative to traditional nosologies. J. Abnorm. Psychol. 126, 454–477. doi: 10.1037/abn0000258, 28333488

[ref52] Kuijper S. J. M. Hartman C. A. Bogaerds-Hazenberg S. T. M. Hendriks P. (2017). Narrative production in children with autism spectrum disorder (ASD) and children with attention-deficit/hyperactivity disorder (ADHD): similarities and differences. J. Abnorm. Psychol. 126, 63–75. doi: 10.1037/abn0000231, 27893232

[ref53] Lancaster H. S. Camarata S. (2019). Reconceptualizing developmental language disorder as a spectrum disorder: issues and evidence. Int. J. Lang. Commun. Disord. 54, 79–94. doi: 10.1111/1460-6984.12433, 30426606 PMC6684235

[ref54] Landa R. Piven J. Wzorek M. M. Gayle J. O. Chase G. A. Folstein S. E. (1992). Social language use in parents of autistic individuals. Psychol. Med. 22, 245–254. doi: 10.1017/S0033291700032918, 1574562

[ref55] Lerner M. D. Calhoun C. D. Mikami A. Y. De Los Reyes A. (2012). Understanding parent–child social informant discrepancy in youth with high functioning autism spectrum disorders. J. Autism Dev. Disord. 42, 2680–2692. doi: 10.1007/s10803-012-1525-9, 22456819

[ref56] Levy F. Hay D. A. McSTEPHEN M. Wood C. Waldman I. (1997). Attention-deficit hyperactivity disorder: a category or a continuum? Genetic analysis of a large-scale twin study. J. Am. Acad. Child Adolesc. Psychiatry 36, 737–744. doi: 10.1097/00004583-199706000-00009, 9183127

[ref57] Lord C. Rutter M. Le Couteur A. (1994). Autism diagnostic interview-revised: a revised version of a diagnostic interview for caregivers of individuals with possible pervasive developmental disorders. J. Autism Dev. Disord. 24, 659–685. doi: 10.1007/BF02172145, 7814313

[ref58] Lord C. Shulman C. DiLavore P. (2004). Regression and word loss in autistic spectrum disorders. J. Child Psychol. Psychiatry 45, 936–955. doi: 10.1111/j.1469-7610.2004.t01-1-00287.x, 15225337

[ref59] Lord C. Storoschuk S. Rutter M. Pickles A. (1993). Using the ADI-R to diagnose autism in preschool children. Infant Ment. Health J. Infancy Early Child. 14, 234–252. doi: 10.1002/1097-0355(199323)14:33C234::AID-IMHJ22801403083E3.0.CO;2-F

[ref60] Loukusa S. Leinonen E. Kuusikko S. Jussila K. Mattila M.-L. Ryder N. . (2007). Use of context in pragmatic language comprehension by children with Asperger syndrome or high-functioning autism. J. Autism Dev. Disord. 37, 1049–1059. doi: 10.1007/s10803-006-0247-2, 17072751

[ref61] Mammarella I. C. Cardillo R. Semrud-Clikeman M. (2022). Do comorbid symptoms discriminate between autism spectrum disorder, ADHD and nonverbal learning disability? Res. Dev. Disabil. 126:104242. doi: 10.1016/j.ridd.2022.104242, 35526491

[ref62] Mammarella I. C. Toffalini E. Caviola S. Colling L. Szűcs D. (2021). No evidence for a core deficit in developmental dyscalculia or mathematical learning disabilities. J. Child Psychol. Psychiatry 62, 704–714. doi: 10.1111/jcpp.13397, 33684972

[ref63] May T. Brignell A. Hawi Z. Brereton A. Tonge B. Bellgrove M. A. . (2018). Trends in the overlap of autism spectrum disorder and attention deficit hyperactivity disorder: prevalence, clinical management, language and genetics. Curr. Dev. Disord. Rep. 5, 49–57. doi: 10.1007/s40474-018-0131-8

[ref64] Meehl P. E. (1995). Bootstraps taxometrics: solving the classification problem in psychopathology. Am. Psychol. 50, 266–275. doi: 10.1037/0003-066X.50.4.266, 7733538

[ref65] Mouti A. Dryer R. Kohn M. (2019). Differentiating autism Spectrum disorder from ADHD using the social communication questionnaire. J. Atten. Disord. 23, 828–837. doi: 10.1177/1087054718781945, 29936891

[ref66] Nilsen E. S. Varghese A. Xu Z. Fecica A. (2015). Children with stronger executive functioning and fewer ADHD traits produce more effective referential statements. Cogn. Dev. 36, 68–82. doi: 10.1016/j.cogdev.2015.09.001

[ref67] Nobile M. Alberti B. Zuddas A. (2007). CRS-R. Conners' Rating Scales Revised. Manuale. Florence: Giunti Editore.

[ref68] Norbury C. F. (2005). The relationship between theory of mind and metaphor: evidence from children with language impairment and autistic spectrum disorder. Br. J. Dev. Psychol. 23, 383–399. doi: 10.1348/026151005X26732

[ref69] Palminteri S. Wyart V. Koechlin E. (2017). The importance of falsification in computational cognitive modeling. Trends Cogn. Sci. 21, 425–433. doi: 10.1016/j.tics.2017.03.011, 28476348

[ref70] Parks K. M. A. Cardy J. E. O. Woynaroski T. G. Sehl C. G. Stevenson R. A. (2021). Investigating the role of inattention and/or hyperactivity/impulsivity in language and social functioning using a dimensional approach. J. Commun. Disord. 89:106036. doi: 10.1016/j.jcomdis.2020.106036, 33249356 PMC8862713

[ref71] Parsons L. Cordier R. Munro N. Joosten A. Speyer R. (2017). A systematic review of pragmatic language interventions for children with autism spectrum disorder. PLoS One 12:e0172242. doi: 10.1371/journal.pone.0172242, 28426832 PMC5398499

[ref72] R Core Team (2024). R: A Language and Environment for Statistical Computing. Vienna: R Core Team.

[ref73] Reindal L. Nærland T. Weidle B. Lydersen S. Andreassen O. A. Sund A. M. (2023). Structural and pragmatic language impairments in children evaluated for autism Spectrum disorder (ASD). J. Autism Dev. Disord. 53, 701–719. doi: 10.1007/s10803-020-04853-1, 33515169 PMC9944009

[ref74] Rints A. McAuley T. Nilsen E. S. (2015). Social communication is predicted by inhibitory ability and ADHD traits in preschool-aged children: a mediation model. J. Atten. Disord. 19, 901–911. doi: 10.1177/1087054714558873, 25477018

[ref75] Roefs A. Fried E. I. Kindt M. Martijn C. Elzinga B. Evers A. W. M. . (2022). A new science of mental disorders: using personalised, transdiagnostic, dynamical systems to understand, model, diagnose and treat psychopathology. Behav. Res. Ther. 153:104096. doi: 10.1016/j.brat.2022.104096, 35500541

[ref76] Ronald A. de B. N. Polderman T. J. C. (2021). Systematic review: how the attention-deficit/hyperactivity disorder polygenic risk score adds to our understanding of ADHD and associated traits. J. Am. Acad. Child Adolesc. Psychiatry 60, 1234–1277. doi: 10.1016/j.jaac.2021.01.019, 33548493 PMC11164195

[ref77] Roselló B. Berenguer C. Navío P. Baixauli I. Miranda A. (2017). Executive functioning, social cognition, pragmatics, and social interaction in attention deficit hyperactivity disorder and autism spectrum disorder. Curr. Dev. Disord. Rep. 4, 72–77. doi: 10.1007/s40474-017-0114-1

[ref78] Ruscio J. Haslam N. Ruscio A. M. (2013). Introduction to the Taxometric Method: A Practical guide. London: Routledge.

[ref79] Ruscio J. Ruscio A. M. (2004). A conceptual and methodological checklist for conducting a taxometric investigation. Behav. Ther. 35, 403–447. doi: 10.1016/S0005-7894(04)80044-3

[ref80] Ruscio J. Walters G. D. Marcus D. K. Kaczetow W. (2010). Comparing the relative fit of categorical and dimensional latent variable models using consistency tests. Psychol. Assess. 22, 5–21. doi: 10.1037/a0018259, 20230147

[ref81] Rutter M. Le Couteur A. Lord C. (2005). ADI-R: Autism Diagnostic Interview–Revised. Florence: Giunti OS Special Organization.

[ref82] Salley B. Gabrielli J. Smith C. M. Braun M. (2015). Do communication and social interaction skills differ across youth diagnosed with autism spectrum disorder, attention-deficit/hyperactivity disorder, or dual diagnosis? Res. Autism Spectr. Disord. 20, 58–66. doi: 10.1016/j.rasd.2015.08.006, 26779281 PMC4709846

[ref83] Shipkova M. Milojevich H. Lindquist K. A. Sheridan M. A. (2025). Children’s emotion word knowledge is associated with adaptive emotion regulation: links to family-level and child-level factors. Emotion 25, 1982–1996. doi: 10.1037/emo0001543, 40424154 PMC12354048

[ref84] Simmons E. S. Paul R. Volkmar F. (2014). Assessing pragmatic language in autism spectrum disorder: the Yale *in vivo* pragmatic protocol. J. Speech Lang. Hear. Res. 57, 2162–2173. doi: 10.1044/2014_JSLHR-L-14-0040 25029348

[ref85] Skuse D. H. Mandy W. Steer C. Miller L. L. Goodman R. Lawrence K. . (2009). Social communication competence and functional adaptation in a general population of children: preliminary evidence for sex-by-verbal IQ differential risk. J. Am. Acad. Child Adolesc. Psychiatry 48, 128–137. doi: 10.1097/CHI.0b013e31819176b8, 19106766

[ref86] Staikova E. Gomes H. Tartter V. McCabe A. Halperin J. M. (2013). Pragmatic deficits and social impairment in children with ADHD. J. Child Psychol. Psychiatry 54, 1275–1283. doi: 10.1111/jcpp.12082, 23682627 PMC3648855

[ref87] Sturrock A. Foy K. Freed J. Adams C. Leadbitter K. (2023). The impact of subtle language and communication difficulties on the daily lives of autistic children without intellectual disability: parent perspectives. Int. J. Lang. Commun. Disord. 58, 1232–1250. doi: 10.1111/1460-6984.12859, 36807949

[ref88] Tager-Flusberg H. Paul R. Lord C. (2005). “Language and communication in autism,” in Handbook of Autism and Pervasive Developmental Disorders, (Oxford: John Wiley & Sons, Ltd).

[ref89] Taylor M. J. Charman T. Ronald A. (2015). Where are the strongest associations between autistic traits and traits of ADHD? Evidence from a community-based twin study. Eur. Child Adolesc. Psychiatry 24, 1129–1138. doi: 10.1007/s00787-014-0666-0, 25600178 PMC4554745

[ref90] Tek S. Mesite L. Fein D. Naigles L. (2014). Longitudinal analyses of expressive language development reveal two distinct language profiles among Young children with autism Spectrum disorders. J. Autism Dev. Disord. 44, 75–89. doi: 10.1007/s10803-013-1853-4, 23719855 PMC4557801

[ref91] Väisänen R. Loukusa S. Moilanen I. Yliherva A. (2014). Language and pragmatic profile in children with ADHD measured by children’s communication checklist 2nd edition. Logop. Phoniatr. Vocology 39, 179–187. doi: 10.3109/14015439.2013.784802 24580020

[ref92] Volden J. Phillips L. (2010). Measuring pragmatic language in speakers with autism spectrum disorders: comparing the children’s communication checklist--2 and the test of pragmatic language. Am. J. Speech Lang. Pathol. 19, 204–212. doi: 10.1044/1058-0360(2010/09-0011) 20220047

[ref93] Wanstall E. A. Doidge J. Weiss J. Toplak M. E. (2019). Estimations of competence in neurodevelopmental conditions: a review. Psihol. Teme 28, 193–232. doi: 10.31820/pt.28.1.10

[ref94] Wechsler D. (2003). Wechsler Intelligence Scale for Children. Agra: Psychological Corporation.

[ref95] Westby C. Watson S. M. R. (2021). “ADHD and communication disorders,” in The Handbook of Language and Speech Disorders, (Oxford: John Wiley & Sons, Ltd).

[ref96] Whyte E. M. Nelson K. E. (2015). Trajectories of pragmatic and nonliteral language development in children with autism spectrum disorders. J. Commun. Disord. 54, 2–14. doi: 10.1016/j.jcomdis.2015.01.001, 25638464

[ref97] Widiger T. A. Samuel D. B. (2005). Diagnostic categories or dimensions? A question for the diagnostic and statistical manual of mental disorders--fifth edition. J. Abnorm. Psychol. 114, 494–504. doi: 10.1037/0021-843X.114.4.494 16351373

[ref98] Wilson R. C. Collins A. G. E. (2019). Ten simple rules for the computational modeling of behavioral data. eLife 8:e49547. doi: 10.7554/eLife.49547, 31769410 PMC6879303

[ref99] Wood S. N. (2017). Generalized Additive Models: An Introduction with R, Second Edition. New York: Chapman and Hall/CRC.

[ref100] World Health Organization (1992). The ICD-10 Classification of Mental and Behavioral Disorders: Clinical Descriptions and Diagnostic Guidelines. Geneva: World Health Organization.

[ref101] Young E. C. Diehl J. J. Morris D. Hyman S. L. Bennetto L. (2005). The use of two language tests to identify pragmatic language problems in children with autism spectrum disorders. Lang. Speech Hear. Serv. Sch. 36, 62–72. doi: 10.1044/0161-1461(2005/006), 15801508

[ref102] Zeidan J. Fombonne E. Scorah J. Ibrahim A. Durkin M. S. Saxena S. . (2022). Global prevalence of autism: a systematic review update. Autism Res. 15, 778–790. doi: 10.1002/aur.2696, 35238171 PMC9310578

[ref103] Zhong Q. Porter M. (2024). Autism spectrum disorder symptoms in individuals with a primary diagnosis of attention-deficit/hyperactivity disorder: a systematic review. Rev. J. Autism Dev. Disord. 13, 424–441. doi: 10.1007/s40489-024-00443-4

