# Supplementary material for: Communication across autism, ADHD, and non-clinical groups: examining autistic and ADHD traits from a dimensional perspective
Source: Front Psychol. 2026 Aug 28;17:1865249. doi: 10.3389/fpsyg.2026.1865249 (PMC13562298; doi:10.3389/fpsyg.2026.1865249)
Supplement: Supplementary file 1 [file Supplementary_file_1.docx]

**Supplementary Materials**

**Additional GAM Plots**

Figure S1 shows the predicted GCC surface from the most flexible hybrid GAM, plotted separately for the ND, ADHD, and ASD groups. In this model, ADHD index and ADI-R were allowed to vary smoothly within each group. Because the ND group occupies a relatively limited portion of the ADHD and ADI-R score ranges, part of the plotted surface for this group reflects extrapolation. The figure is therefore provided for descriptive purposes only. The predicted GCC surface from the final trait-only GAM is reported in the main text.

**Figure S1**

*Predicted GCC values from the flexible hybrid GAM, plotted separately for ND, ADHD, and ASD groups.*

*Note. GCC = Global Communication Composite; ADHD index = ADHD traits; ADI-R = autism traits. In the ND panel, part of the surface extends beyond the observed range of ADHD and ADI-R scores and should be interpreted as extrapolation.*

**Measurement checks for the GCC and communication subcomponents**

Because the GCC combines structural and pragmatic aspects of communication, we conducted additional measurement checks to evaluate whether these two components provided clearly separable outcome dimensions in the present data. A two-factor CFA was fitted in the ND group, with structural language items loading on one factor and pragmatic communication items loading on a second factor. Model fit was acceptable according to CFI and SRMR, but RMSEA was elevated, CFI = .949, TLI = .925, RMSEA = .108, SRMR = .047. The latent correlation between the structural and pragmatic factors was high, r = .827. Internal consistency was also high for the full GCC composite, alpha = .923, and for the structural and pragmatic composites separately, alpha = .851 and alpha = .908, respectively.

These results suggest that the structural and pragmatic composites were reliable but strongly associated. For this reason, the GCC was retained as the primary outcome. Separate analyses for structural and pragmatic composites were treated as sensitivity checks, rather than as a basis for interpreting domain-specific communication profiles.

**Additional GAM model comparisons and sensitivity analyses**

**Main model comparison**

Table S1 reports the main GAM model comparisons. The group-only model showed substantially poorer fit than models including continuous trait scores. The lowest BIC was obtained by the trait-only model including smooth terms for ADHD index and ADI-R, with no diagnostic group predictor.

**Table S1**

Main GAM model comparisons for GCC

| **Model** | **Predictors** | **BIC** | **Adjusted R^2^** |
| --- | --- | --- | --- |
| Group-only | Group | 4324.75 | 0.34 |
| Additive hybrid | Group + s(ADHD index) + s(ADI-R) | 4163.31 | 0.55 |
| Flexible hybrid | s(ADHD index, by=Group) + s(ADI-R) | 4159.74 | 0.55 |
| Trait-only | s(ADHD index) + s(ADI-R) | 4151.39 | 0.55 |

*Note. GCC = Global Communication Composite. ADHD index = ADHD traits. ADI-R = autism traits. The flexible hybrid model was selected as the best-fitting hybrid variant from a set of six candidate models combining group and trait predictors in different configurations (BIC range: 4159.74-4186.50). Lower BIC indicates better balance between fit and complexity.*

**Sensitivity analyses**

We conducted additional sensitivity analyses to evaluate whether the main pattern was robust to covariate adjustment, alternative outcome definitions, and reduced overlap between the ADI-R predictor and GCC outcome.

**Table S2**

Sensitivity analyses

| **Model** | **Predictors** | **BIC** | **Adjusted R²** |
| --- | --- | --- | --- |
| Age- and sex-adjusted model | s(ADHD index) + s(ADI-R) + s(age) + sex | 4140.01 | 0.57 |
| Age-, sex-, and IQ-adjusted model | s(ADHD index) + s(ADI-R) + s(age) + sex + s(IQ) | 4143.52 | 0.58 |
| Non-communication ADI-R model | s(ADHD index) + s(ADI-R non-communication composite) | 4172.78 | 0.53 |
| Structural communication, group-only | Group | 3736.78 | 0.18 |
| Structural communication, trait-only | s(ADHD index) + s(ADI-R) | 3616.62 | 0.38 |
| Pragmatic communication, group-only | Group | 3699.13 | 0.43 |
| Pragmatic communication, trait-only | s(ADHD index) + s(ADI-R) | 3553.45 | 0.60 |

*Note. The age- and sex-adjusted model improved BIC relative to the primary trait-only model, but did not change the substantive interpretation. Adding IQ did not further improve BIC relative to the age- and sex-adjusted model. The model using the non-communication ADI-R composite showed poorer fit than the primary model, but still fitted substantially better than the group-only model. Separate analyses for structural and pragmatic communication composites reproduced the same broad pattern as the GCC analysis, but were interpreted as sensitivity checks because the two composites were strongly associated.*

**GAM diagnostics**

We inspected standard GAM diagnostics for the final trait-only model. The model showed limited non-linearity: the ADHD smoother used 2.04 effective degrees of freedom and the ADI-R smoother used 3.33 effective degrees of freedom. Basis-dimension checks did not indicate that the chosen basis dimension was too low, with k-index values close to 1 for both smoothers. Concurvity was low in the final trait-only model, with estimated concurvity between the two smooth terms below .18 (observed index = .23). These diagnostics suggested that the final model was not driven by excessive wiggliness or unstable smoother estimation.

**Table S3**

Basis-dimension checks for the final trait-only GAM

| **Smoother** | **k'** | **edf** | **k-index** | **p** |
| --- | --- | --- | --- | --- |
| s(ADHD index) | 9 | 2.04 | 0.97 | .25 |
| s(ADI-R) | 9 | 3.33 | 0.97 | .26 |

*Note. edf = effective degrees of freedom. Values of the k-index close to 1 did not indicate that the chosen basis dimension was too low*

We also checked whether results depended on the chosen basis dimension. BIC values were very similar across k = 5, 10, 15, and 20, and the estimated degrees of freedom remained stable. This indicates that the main result was not sensitive to the specific basis dimension used in the primary model.

Table S4

Sensitivity to basis dimension

| **Basis dimension** | **BIC** | **edf for ADHD index** | **edf for ADI-R** |
| --- | --- | --- | --- |
| k = 5 | 4149.39 | 2.01 | 3.00 |
| k = 10 | 4151.39 | 2.04 | 3.33 |
| k = 15 | 4151.52 | 2.05 | 3.35 |
| k = 20 | 4151.56 | 2.05 | 3.36 |

*Note. BIC values and effective degrees of freedom were stable across alternative basis dimensions, indicating that the main result was not sensitive to the specific basis dimension used in the primary model.*

**Simulation-based evaluation of the model-selection procedure**

The main analyses compared prespecified categorical, trait-only, and hybrid GAMs using BIC. Because diagnostic group labels and continuous trait measures are strongly related by design, hybrid models may display concurvity and can be difficult to interpret. We therefore conducted a simulation-based evaluation of the model-selection procedure. The aim was not to test the latent structure of ASD or ADHD, and the simulations should not be interpreted as validating a dimensional or categorical ontology. The aim was narrower: to examine whether the same GAM-BIC procedure used in the empirical analyses could recover trait-driven, group-driven, and hybrid patterns under conditions resembling the empirical sample.

The simulations were intentionally calibrated to the present dataset. They mirrored the empirical group sizes, with ND = 241, ADHD = 105, and ASD = 147. ADHD and ASD trait scores were generated from group-specific normal distributions approximating the empirical means and standard deviations, and were truncated to the observed scale ranges, ADHD index = 30-100 and ADI-R = 0-63. GCC values were generated with Gaussian noise and truncated to the observed 0-140 range.

Before the main simulation study, we checked calibration using very large synthetic samples, with n = 10,000 per group. This step ensured that simulated group differences and trait-outcome associations were similar to those observed in the empirical data, avoiding unrealistically easy model-recovery conditions. In the trait-driven scenario, GCC correlated with ADHD traits at r = -.47 and with ASD traits at r = -.65, and the ND-ADHD and ND-ASD standardized mean differences were approximately d = 1.03 and d = 1.63. In the group-driven scenario, the corresponding trait-GCC correlations were weaker, r = -.24 and r = -.40, while group differences remained similar, d = 1.00 and d = 1.63. In the hybrid scenario, trait-GCC correlations were intermediate, r = -.36 and r = -.54, and group differences were d = 1.08 and d = 1.74.

**Data-generating scenarios**

Trait-driven scenario. GCC varied as a function of continuous ADHD and ASD trait scores, with no direct group effect on the outcome. ADHD and ASD traits were moderately correlated. Trait effects included mild non-linearity, producing a gradual flattening at higher trait values.

Group-driven scenario. GCC differed by diagnostic group, with fixed shifts for ADHD and ASD relative to ND. Trait effects on GCC were set to zero in expectation. Associations between trait scores and GCC arose indirectly because trait distributions differed across diagnostic groups.

Hybrid scenario. GCC was influenced by both diagnostic group and continuous ADHD and ASD trait scores. This scenario combined group-level shifts with trait effects and mild non-linearity, with the two sources weighted equally. Other mixing proportions could yield different recovery rates, and the present results reflect a single operationalization of a hybrid pattern calibrated to the empirical sample.

**Models fitted to each simulated dataset**

For each simulated dataset, we fitted four GAMs corresponding to the candidate model families used in the empirical analyses:

1. Group-only model: GCC ~ Group

2. Trait-only model: GCC ~ s(ADHD) + s(ASD)

3. Additive hybrid model: GCC ~ Group + s(ADHD) + s(ASD)

4. Flexible hybrid model: GCC ~ Group + s(ADHD, by = Group) + s(ASD, by = Group)

BIC was computed for all four models. For each replication, we recorded the model with the lowest BIC and the ΔBIC between the best and second-best models.

**Simulation results**

Across 1,000 replications per scenario, the GAM-BIC procedure generally recovered the expected model family. Recovery was strongest in the trait-driven and group-driven scenarios, and lower in the hybrid scenario, as expected because hybrid patterns are more difficult to distinguish from simpler trait-only or group-only patterns.

**Table S5**

Model recovery across 1,000 replications per scenario

| **Scenario** | **Expected model** | **Recovery** | **Recovery with ΔBIC > 2** | **Recovery with ΔBIC > 6** | **Recovery with ΔBIC > 10** | **Median ΔBIC** |
| --- | --- | --- | --- | --- | --- | --- |
| Trait-driven | Trait-only | .988 | .981 | .918 | .657 | 10.85 |
| Group-driven | Group-only | .998 | .997 | .982 | .868 | 12.46 |
| Hybrid | Additive hybrid | .870 | .797 | .653 | .510 | 10.58 |

*Note. Recovery indicates the proportion of replications in which the expected model had the lowest BIC. ΔBIC is the difference between the best and second-best models. Hybrid patterns were less clearly recovered than trait-driven or group-driven patterns, as expected when group and trait information overlap.*

In the trait-driven scenario, the expected trait-only model was selected in 98.8% of replications. In the group-driven scenario, the expected group-only model was selected in 99.8% of replications. In the hybrid scenario, the expected additive hybrid model was selected in 87.0% of replications. Most errors in the hybrid scenario involved selection of the trait-only model, indicating that hybrid effects can be partially absorbed by continuous trait smoothers when group and trait information overlap.

Overall, the simulations indicate that, under conditions similar to those observed in the empirical data, the GAM-BIC procedure can usually distinguish trait-driven, group-driven, and hybrid patterns. However, hybrid patterns are less clearly recovered than purely trait-driven or group-driven patterns. The simulation results should therefore be interpreted as a targeted check of the model-selection procedure under realistic conditions, not as a general validation of the analytic strategy or as evidence about the latent structure of ASD or ADHD.

**Illustration of the trait-driven data-generating process**

Figures S2-S4 show one example dataset generated under the trait-driven scenario. These figures illustrate the joint ADHD-ASD trait distribution and the expected GCC functions across selected values of the other trait. The plots show the mild non-linearity built into the data-generating process and the realistic degree of overlap between groups.

**Figure S2**

*Example simulated dataset under the trait-driven scenario: ADHD and ASD trait scores by group.*

**Figure S3**

*Example simulated dataset under the trait-driven scenario: GCC as a function of ADHD traits, plotted at selected ASD trait levels.*

**Figure S4**

*Example simulated dataset under the trait-driven scenario: GCC as a function of ASD traits, plotted at selected ADHD trait levels.*
